# Aggrephagy-related patterns in tumor microenvironment, prognosis, and immunotherapy for acute myeloid leukemia: a comprehensive single-cell RNA sequencing analysis

**DOI:** 10.3389/fonc.2023.1195392

**Published:** 2023-07-17

**Authors:** Yan Pan, Yingjian Wang, Mengsi Hu, Shoufang Xu, Feiyu Jiang, Yetao Han, Fangjian Chen, Zhiwei Liu

**Affiliations:** ^1^Department of Blood Transfusion, The Quzhou Affiliated Hospital of Wenzhou Medical University, Quzhou People’s Hospital, Quzhou, Zhejiang, China; ^2^Department of Blood Transfusion, Sir Run Run Shaw Hospital, Zhejiang University School of Medicine, Hangzhou, Zhejiang, China

**Keywords:** acute myeloid leukemia, aggrephagy, immune cell, prognosis, immunotherapy, microenvironment

## Abstract

Acute myeloid leukemia (AML) is a complex mixed entity composed of malignant tumor cells, immune cells and stromal cells, with intra-tumor and inter-tumor heterogeneity. Single-cell RNA sequencing enables a comprehensive study of the highly complex tumor microenvironment, which is conducive to exploring the evolutionary trajectory of tumor cells. Herein, we carried out comprehensive analyses of aggrephagy-related cell clusters based on single-cell sequencing for patients with acute myeloid leukemia. A total of 11 specific cell types (T, NK, CMP, Myeloid, GMP, MEP, Promono, Plasma, HSC, B, and Erythroid cells) using t-SNE dimension reduction analysis. Several aggrephagy-related genes were highly expressed in the 11 specific cell types. Using Monocle analysis and NMF clustering analysis, six aggrephagy-related CD8^+^ T clusters, six aggrephagy-related NK clusters, and six aggrephagy-related Mac clusters were identified. We also evaluated the ligand-receptor links and Cell–cell communication using CellChat package and CellChatDB database. Furthermore, the transcription factors (TFs) of aggrephagy-mediated cell clusters for AML were assessed through pySCENIC package. Prognostic analysis of the aggrephagy-related cell clusters based on R package revealed the differences in prognosis of aggrephagy-mediated cell clusters. Immunotherapy of the aggrephagy-related cell clusters was investigated using TIDE algorithm and public immunotherapy cohorts. Our study revealed the significance of aggrephagy-related patterns in tumor microenvironment, prognosis, and immunotherapy for AML.

## Introduction

Leukemia is a malignant clonal disease originating from hematopoietic stem cells (1). The affected cells have uncontrolled proliferation, impaired differentiation, and blocked apoptosis, so the affected cells are stuck in different stages of cell development (2). The incidence and mortality rate of leukemia are both high. The report showed that in 2018 alone, there were 437000 new cases of leukemia and 309000 new deaths from leukemia worldwide (3).

Leukemia can be classified as acute (4) or chronic (5) according to its course. Leukemias can be divided into myeloid leukemia and lymphocytic leukemia according to the cells involved (6). Acute myeloid leukemia (AML), the most common leukemia in adults, is a highly heterogeneous disease (7). French-American-British (FAB) defined eight subtypes (M0 to M7) based on the morphological and cytological characteristics of leukemia cells (8). According to genetics, morphology, immunophenotype and clinical manifestations, World Health Organization (WHO) classified leukemia into six main types and more than 20 subtypes (9). In addition, the prognosis of AML can be divided into good, moderate and poor groups based on cytogenetic characteristics (1, 6), but the prognosis of different patients in each group is still very different, indicating that the gene expression pattern of leukemia is very complex.

Tumor microenvironment (TME) is the internal environment that tumor cells depend on for survival and development. Besides tumor cells, it also contains many non-malignant cells and some soluble factors, which play an important role in promoting tumor occurrence, progression and immune escape (10). Tumor microenvironment mainly includes immune microenvironment, including myeloid-derived suppressor cells (MDSCs), tumor-associated macrophages (TAMs), tumor-associated neutrophils (TANs), dendritic cell (DC), T cell, B cell, and Natural Killer (NK) cell, and non-immune microenvironment, including cancer-associated fibroblasts (CAFs), extracellular matrix, mesenchymal stem cells, and various secreted factors (11–14). Therefore, tumor is a complex mixed entity composed of malignant tumor cells, immune cells and stromal cells, with intra-tumor and inter-tumor heterogeneity. Since bulk tissue is composed of various cells, its sequencing cannot reveal the function or cell state of a specific cell population (15). Therefore, the detection of genome, transcriptome, epigenome and proteome at the cellular level can overcome the limitations of the traditional bulk level and conduct more detailed analysis at the cellular and molecular level (15). Single-cell RNA sequencing (scRNA-Seq) enables non-targeted quantification of transcripts in a single cell. Single-cell RNA sequencing enables a comprehensive study of the highly complex tumor microenvironment, which is conducive to exploring the evolutionary trajectory of tumor cells, the complex interactions between tumor cells and tumor microenvironment, and the spatio-temporal functional relationships between different cell population types (16, 17). Bioinformation analysis can identify new cell types, identify rare cell populations, and construct cell status and phylogenetic maps through computational methods such as high-dimensional data reduction, unsupervised clustering, phylogenetic modeling, locus inference, RNA rate analysis, lineage tracing, and ligand-receptor interaction mapping (16, 17).

Autophagy is an important feedback process of cells under pressure. Autophagy realizes self-digestion and catabolism by phagocytic organelles and degradation of cell contents, so as to maintain the homeostasis balance of cells (18, 19). Autophagy plays an important role in maintaining vital activities and immune function and is closely related to tumors and other diseases. The common types of autophagy include macroautophagy, microautophagy and chaperonemediated autophagy (20). Aggrephagy is a kind of selective autophagy, which is the only way to clear protein aggregates. Once the function of molecular chaperone and ubiquitin proteasome is limited or the clearance efficiency of misfolded proteins is lower than the production rate, protein aggregates will be formed, and the aggrephagy needs to be activated to degrade them (21).

In this study, the relationship between aggrephagy-related genes and cell subsets of TME (such as T cells, Natural Killer cell, and Myeloid cells) for AML was investigated using data of single-cell RNA-sequencing (scRNA-seq) from GSE116256. After Nonnegative Matrix Factorization analysis, the characteristics of the aggrephagy-mediated cell clusters in Pseudotime trajectory, cell–cell communication, ligand-receptor links, and immunotherapy were investigated.

## Materials and methods

### Downloading and preprocessing for data of acute myeloid leukemia

The samples source with single-cell RNA-sequencing (scRNA-seq, GSE116256) and expression profiles (GSE63270 and GSE12417) were downloaded from Gene Expression Omnibus (GEO) database (https://www.ncbi.nlm.nih.gov/geo/) of The National Center for Biotechnology Information (NCBI) (22). We enrolled three normal samples and ten patients with acute myeloid leukemia from GSE116256 for analysis of scRNA-seq (23–25). There were 104 normal and acute myeloid leukemia (42 populations and 62 leukemic populations) included in GSE63270 dataset (26). GSE12417 dataset contained the analysis of 79 samples of bone marrow or peripheral blood mononuclear cells from adult patients with untreated acute myeloid leukemia (27, 28). In addition, the expression profiles and clinical information were acquired from TCGA-LAML cohort, including 151 patients with acute myeloid leukemia (29, 30).

### Dimensionality reduction and annotation of single cell for acute myeloid leukemia

First, the data of single cell was filtered by setting each gene to be expressed in at least three cells, and each cell to express at least 500 genes, resulting in 9891 cells. We calculated the percentage of mitochondria and Ribosomal RNA (rRNA) through the PercentageFeatureSet function of Seurat package (31). The number of genes expressed in each single cell was greater than 100 and less than 5000, and we ensured the percentage of mitochondria was less than 20%. Furthermore, the Unique Molecular Identifier (UMI) of the single cell was at least greater than 100, resulting in 9886 cells. Subsequently, we used the method of log-normalization to standardize the single-cell data from each of the 13 samples. The highly variable features were identified by FindVariableFeatures function (32) based on variance stabilization transformation (VST). The genes were then scaled by using the ScaleData function for all genes. We utilized RunPCA function for PCA dimension reduction to find anchors. The FindNeighbors function with dim=15 and FindClusters function with Resolution=0.1 was used to luster cells. Ulteriorly, the RunTSNE function was used to conduct t-SNE (T-Distribution Stochastic Neighbour Embedding) dimension reduction analysis and the RunUMAP function was used to conduct UMAP (Uniform Manifold Approximation and Projection) reduction analysis. The marker genes for single cell were supplied by SingleR package (33) and the classical marker from the published literature (25).

### Pseudotime trajectory analysis for the aggrephagy-mediated cell clusters

Monocle R package was applied for the data of single cell to explore the correlation of aggrephagy-related genes and pseudotime trajectories (34). The graphs for the pseudotime trajectories of specific cell with aggrephagy-related genes were plotted using the function from Monocle R package, such as plot_pseudotime_heatmap and so on.

### Nonnegative matrix factorization of aggrephagy-related genes in single cell for acute myeloid leukemia

Based on the expression matrix of the scRNA-seq, dimension reduction analysis of aggrephagy-related genes in each cell clusters were conducted employing NMF (Nonnegative Matrix Factorization) R package (35, 36), thus displaying the effect of aggrephagy-related genes in single cell for acute myeloid leukemia.

### Identifying the marker genes of single cell for acute myeloid leukemia

FindAllMarkers function was applied to identify he marker genes of single cell for acute myeloid leukemia (31). The aggrephagy-mediated cell clusters were identified based on differentially expressed genes (DEGs) with log Fold Change (logFC) and aggrephagy-related genes. The NK cell subtypes were summarized from the published literature of Huan Liu et al (37).

### Analysis of transcription factors for aggrephagy-mediated cell clusters

SCENIC was a tool for simultaneously reconstructing gene regulatory networks and identifying stable cell states from single-cell RNA-seq data (38). The gene regulatory network was inferred based on co-expression and DNA motif analysis, and then network activity was analyzed in each cell to identify cell status (38). We carried out analysis of transcription factors (TFs) for aggrephagy-mediated cell clusters for acute myeloid leukemia through pySCENIC package (39–41). RcisTarget R package and two gene-motif rankings (hg19-tss-centered-10 kb and hg19-500 bp-upstream) was used to identify binding motifs of TFs in the gene list for acute myeloid leukemia (42, 43). The threshold value for the TFs was set as Benjamini–Hochberg false discovery rate (BH-FDR) <0.05.

### Cell–cell communication analysis among cell subsets for acute myeloid leukemia

The signaling inputs and outputs among the cell types and aggrephagy-mediated cell clusters were assessed by applying CellChat package (44) and CellChatDB database (45). The netVisual_circle function was utilized for evaluating the strength of cell–cell communication networks among cell subsets (44, 45). In addition, the ligand-receptor interactions among the specific cell subsets were estimated *via* the netVisual_bubble function (44, 45).

### Prognostic analysis of the aggrephagy-related cell clusters for acute myeloid leukemia

Based on the data of scRNA, Gene Set Variation Analysis (GSVA) was applied to compute the signature scores involved in aggrephagy for public database (46). We carried out Cox proportional hazard regression to evaluate the prognosis for the aggrephagy-related cell clusters (47). The Kaplan–Meier curves was plotted through the survminer R package.

### Immunotherapy analysis of the aggrephagy-related cell clusters for acute myeloid leukemia

We used TIDE (Tumor Immune Dysfunction and Exclusion) algorithm to analyze the immune checkpoint blockade immunotherapeutic for the aggrephagy-related cell clusters (48). We also reviewed the published literature to validate the prognostic and therapeutic effects of each cell subtype using real-world immunotherapy cohorts (49–60).

### Statistical analysis

The continuous or category variables were compared using Student’s t-test, Wilcoxon rank sum test, Kruskal–Wallis’s test, or Chi-square test. The log-rank test was used for survival analyses.

## Results

### Dimensionality reduction and annotation of single cell for acute myeloid leukemia

**Figure 1:**
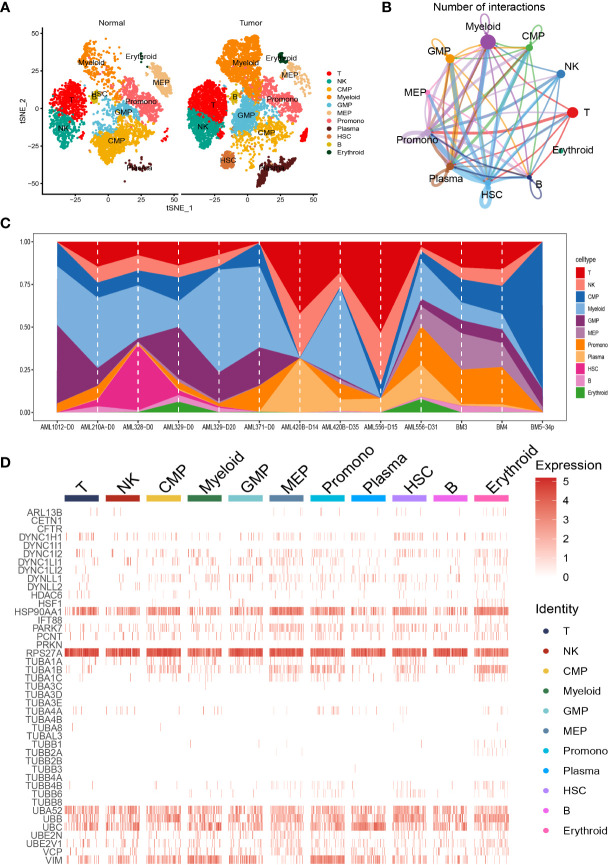
Dimensionality reduction and annotation of single cell for acute myeloid leukemia. **(A)** Cells were clustered into 11 specific cell types using t-SNE algorithm. **(B)** The number of interactions for communication among the 11 specific cell types. **(C)** The proportion of the 11 specific cell types in each sample. **(D)** Heat map showing the expression of the aggrephagy-related genes in 11 specific cell types.

We carried out dimensionality reduction and annotation of single cell for acute myeloid leukemia as described in the *materials and methods* section. We ensured that the number of genes expressed in each single cell was greater than 100 and less than 5000, the percentage of mitochondria was less than 20%, and the Unique Molecular Identifier (UMI) of the single cell was at least greater than 100, resulting in 9886 cells. **Supplementary Figures S1A–B** was the statistical diagram of cell filtration, which could be seen to meet all thresholds set above (**Supplementary Figures S1A, B**). The highly variable features were identified *via* FindVariableFeatures function based on VST, and the top ten highly variable genes among the single cell were marked out in the volcano plot, including IGLL5, HBB, JCHAIN, HBG1, HBG2, HBD, HBA2, CLC, CA1, and HBA1 (**Supplementary Figure S1C**). Ulteriorly, PCA analysis was carried out on the highly variable genes, we used the Elbow algorithm to carry out the Standard Deviation based on the highly variable genes (**Supplementary Figure S1D**). The RunTSNE function was used to conduct t-SNE dimension reduction analysis and the RunUMAP function was used to conduct UMAP reduction analysis, thus identifying a total of 18 cell subsets (**Supplementary Figure S1E**). Afterwards, the marker genes were used to annotate the specific cell types, thus identifying 11 specific cell types, including T cell, Natural Killer (NK) cell, Common Myeloid Progenitor (CMP) cell, Myeloid cell, Granulocyte Monocyte Progenitor (GMP) cell, Megakaryocyte Erythroid Progenitor (MEP) MEP, Promonocyte (Promono) cell, Plasma cell, Hematopoietic Stem Cell (HSC) cell, B cell, and Erythroid cell (**Figure 1A**). We plotted the correlation network for the number of interactions among the 11 specific cell types (**Figure 1B**). **Figure 1C** visually showed the proportion of different cell types in each sample. Finally, we created the heat map to show the expression of the aggrephagy-related genes in different cell types (**Figure 1D**). We could see that there were several aggrephagy-related genes that were highly expressed in the 11 specific cell types, including HSP90AA1, RPS27A, UBA52, UBB, UBC, and VIM (**Figure 1D**). We displayed the global view of the expression pattern for marker genes gained as described in the methods section, reflecting the dynamic features of each cell subsets (**Supplementary Figure S2**).

### Pseudotime analysis for aggrephagy-mediated T cells

**Figure 2:**
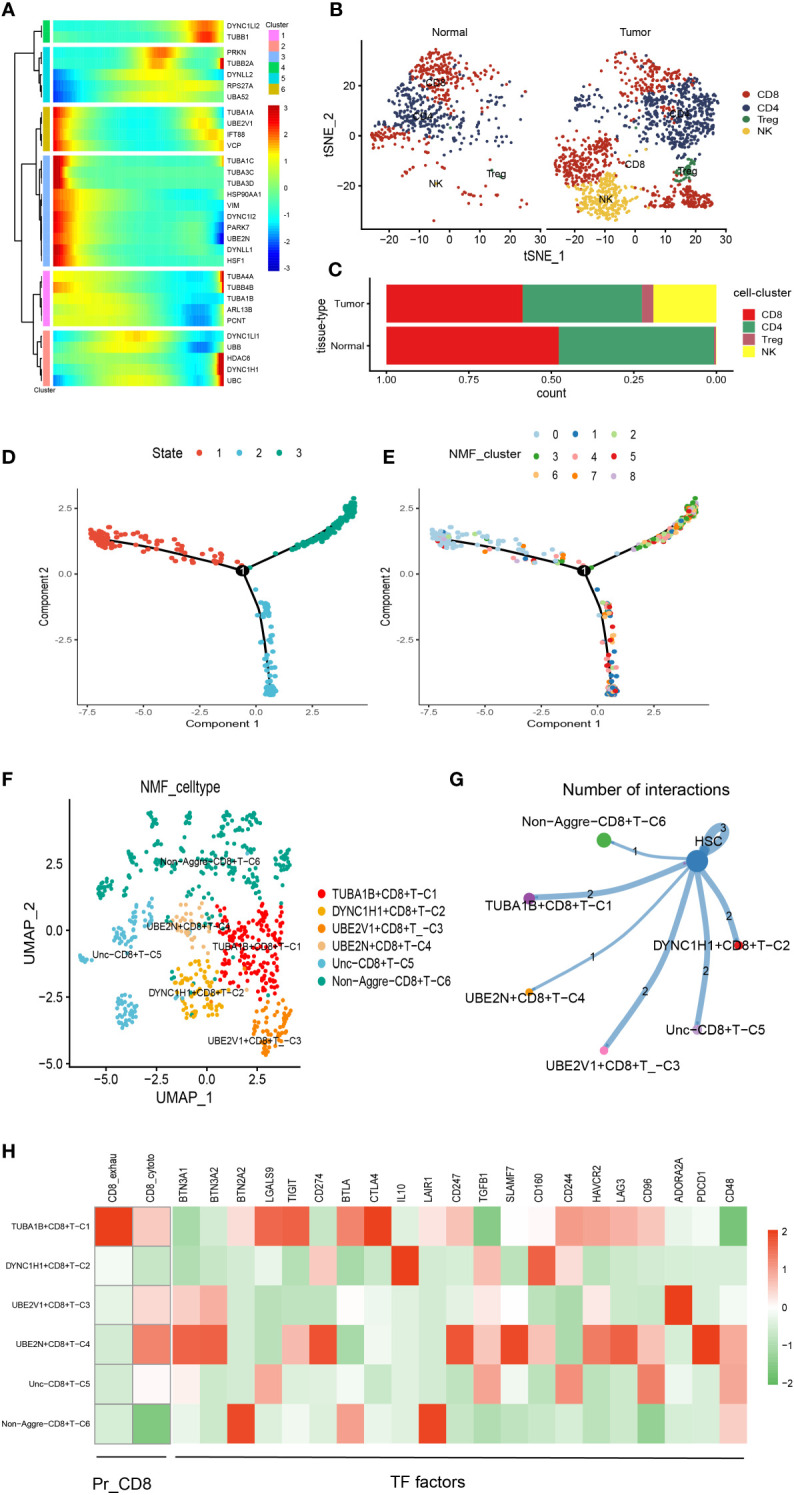
Pseudotime analysis for aggrephagy-mediated T cells. **(A)** Pseudotime analysis reveals the role of aggrephagy-related genes for T cells (2397 cells). **(B)** Four subgroups of T cells were obtained by re-clustering annotation based on tSNE analysis. **(C)** Bar plot showing the percentage of the four cell subgroups (CD8^+^ T cell, CD4^+^ T cell, NK cell and Treg cell). **(D)** Trajectory color-coded by cell state. **(E)** Trajectory color-coded by NMF cluster. **(F)** The UMAP view and clustering analysis identifying six aggrephagy-related CD8^+^ T clusters. **(G)** Cell–Cell communications from aggrephagy-related CD8^+^ T cells to HSC cell. **(H)** Heatmap showing the different average expression of exhausted CD8^+^ T (CD8^+^_exhau), cytotoxic CD8^+^ T (CD8^+^_cyoto), and TFs among the six aggrephagy-related CD8^+^ T clusters.

There was a total of 2397 cells in the T cell type. Using UMAP reduction analysis, the 2397 cells in the T cell type could be clustered into eight cell clusters (**Supplementary Figure S3**), and the global view of the expression pattern for marker genes of the eight cell clusters was displayed in **Supplementary Figure S3**. Further, the eight cell clusters could be re-clustered into nine cell subsets (**Supplementary Figure S3**). Based on the aggrephagy-related genes, six clusters were identified using Monocle analysis (**Figure 2A**), including Cluster 1 (DYNC1LI2 and TUBB1), Cluster 2 (PRKN, TUBB2A, DYNLL2, RPS27A and UBA52), Cluster 3 (TUBA1A, UBE2V1, IFT88 and VCP), Cluster 4 (TUBA1C, TUBA3C, TUBA3D, HSP90AA1, VIM, DYNC1I2, PARK7, UBE2N, DYNLL1 and HSF1), Cluster 5 (TUBA4A, TUBB4B, TUBA1B, ARL13B and PCNT), and Cluster 6 (DYNC1LI1, UBB, HDAC6, DYNC1H1 and UBC). From the heatmap generated by Pseudotime analysis, the critical role of the aggrephagy-related genes in the trajectory process of T cells was observed (**Figure 2A**). Four subgroups of T cells were obtained by re-clustering annotation using t-SNE dimension reduction analysis, including CD8^+^ T cell, CD4^+^ T cell, natural killer (NK) cells, and Regulatory T (Treg) cells (**Figure 2B**). Among the four cell subgroups, we found that CD8^+^ T cell and CD4^+^ T cell had a higher percentage both in tumor samples and normal samples than the other two cell subgroups (**Figure 2C**), and CD8^+^ T cell occupied the highest proportion among the four cell subgroups (**Figure 2C**). The result of CD8^+^ T cell revealed that the whole trajectory could be divided into three segments (State 1, State 2, and State 3) on the basis of the developmental order (**Figure 2D**). Ulteriorly, NMF clustering analysis of the aggrephagy-related gene set for the trajectories showed that these cells aggregated into nine clusters (**Figure 2E**). In addition, the results of UMAP reduction analysis indicated that the NMF cell types were clustered into six aggrephagy-related CD8^+^ T clusters, including TUBA1B+CD8^+^ T−C1, DYNC1H1+CD8^+^ T−C2, UBE2V1+CD8^+^ T−C3, UBE2N+CD8^+^ T−C4, Unc−CD8^+^ T−C5, and Non−Aggre−CD8^+^ T−C6 (**Figure 2F**). The number of ligand-receptor links among the six aggrephagy-related CD8+ T clusters was computed by Cell-Chat analysis (**Figure 2G**). The weights and strength of ligand-receptor links among the six aggrephagy-related CD8^+^ T clusters was computed by Cell-Chat analysis (**Supplementary Figure S3**). Lastly, the discrepancies in the exhausted CD8^+^ T (CD8^+^_exhau), cytotoxic CD8^+^ T (CD8^+^_cyoto), and TFs (BTN3A1, BTN3A2, BTN2A2, LGALS9, TIGIT, CD274, BTLA, CTLA4, IL10, LAIR1, CD247, TGFB1, SLAMF7, CD160, CD244, HAVCR2, LAG3, CD96, ADORA2A, PDCD1, and CD48) among the six aggrephagy-related CD8^+^ T clusters were visually displayed in the pathway heatmap (**Figure 2H**). TUBA1B+CD8^+^ T−C1 tended to be exhausted CD8^+^ T (CD8^+^_exhau), while UBE2N+CD8^+^ T−C4 tended to be cytotoxic CD8^+^ T (CD8^+^_cyoto) as shown in **Figure 2H**. It is noteworthy that TFs of LGALS9, TIGIT, BTLA, and CTLA4 were upregulated in the TUBA1B+CD8^+^ T−C1, TFs of IL10 and CD160 were upregulated in the DYNC1H1+CD8^+^ T−C2, ADORA2A was upregulated in the UBE2V1+CD8^+^ T−C3, BTN3A1, BTN3A2, CD274, CD247, SLAMF7, LAG3, and PDCD1 were upregulated in the UBE2N+CD8^+^ T−C4, BTN2A2 and LAIR1 were upregulated in the Non− Aggre−CD8^+^ T−C6 (**Figure 2H**).

### Pseudotime analysis for aggrephagy-mediated NK cells

**Figure 3:**
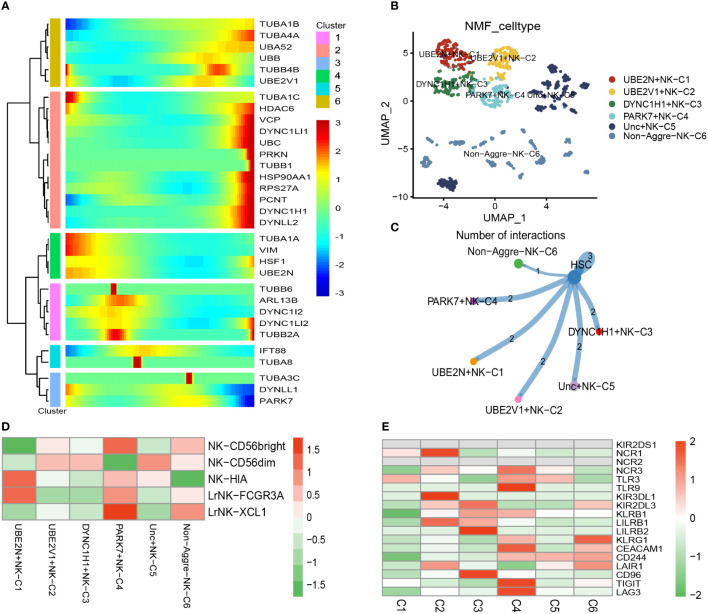
Pseudotime analysis for aggrephagy-mediated NK cells. **(A)** Pseudotime analysis reveals the role of aggrephagy-related genes for NK cells (1067 cells). **(B)** The UMAP view and clustering analysis identifying six aggrephagy-related NK clusters. **(C)** Cell–Cell communications from aggrephagy-related NK cells to HSC cell. **(D)** Heatmap showing the different average expression of NK−CD56bright, NK−CD56dim, NK−HIA, LrNK−FCGR3A, and LrNK−XCL1 among the six aggrephagy-related NK clusters. **(E)** Heatmap showing the different average expression of KIR2DS1, NCR1, NCR2, NCR3, TLR3, TLR9, KIR3DL1, KIR2DL3, KLRB1, LILRB1, LILRB2, KLRG1, CEACAM1, CD244, LAIR1, CD96, TIGIT, and LAG3 among the six aggrephagy-related NK clusters.

There was a total of 1067 cells in the NK cell type. Using UMAP reduction analysis, the 1067 cells in the NK cell type could be clustered into eleven cell clusters (**Supplementary Figure S4**). Based on the aggrephagy-related genes, six clusters were identified using Monocle analysis (**Figure 3A**), including Cluster 1 (TUBA1B, TUBA4A, UBA52, UBB, TUBB4B, and UBE2V1), Cluster 2 (TUBA1C, HDAC6, VCP, DYNC1LI1, UBC, PRKN, TUBB1, HSP90AA1, RPS27A, PCNT, DYNC1H1, and DYNLL2), Cluster 3 (TUBA1A, VIM, HSF1, and UBE2N), Cluster 4 (TUBB6, ARL13B, DYNC1I2, DYNC1LI2, and TUBB2A), Cluster 5 (IFT88 and TUBA8), Cluster 6 (TUBA3C, DYNLL1, and PARK7). The results of UMAP reduction analysis indicated that the NMF cell types were clustered into six aggrephagy-related NK clusters, including UBE2N+NK−C1, UBE2V1+NK−C2, DYNC1H1+NK−C3, PARK7+NK−C4, Unc+NK−C5, and Non−Aggre−NK−C6 (**Figure 3B**). The number of ligand-receptor links among the six aggrephagy-related NK clusters was computed by Cell-Chat analysis (**Figure 3C**). The number, weights and strength of ligand-receptor links among the aggrephagy-related NK clusters was computed by Cell-Chat analysis (**Supplementary Figure S4**). Lastly, the discrepancies in the NK−CD56bright, NK−CD56dim, NK−HIA, LrNK−FCGR3A, LrNK−XCL1, KIR2DS1, NCR1, NCR2, NCR3, TLR3, TLR9, KIR3DL1, KIR2DL3, KLRB1, LILRB1, LILRB2, KLRG1, CEACAM1, CD244, LAIR1, CD96, TIGIT, and LAG3 among the six aggrephagy-related NK clusters were visually displayed in the pathway heatmap (**Figures 3D, E**). It is noteworthy that NK−CD56bright was upregulated in PARK7+NK−C4 (**Figure 3D**), NK−HIA was upregulated in UBE2N+NK−C1 (**Figure 3D**), LrNK−FCGR3A was upregulated in UBE2N+NK−C1 (**Figure 3D**), LrNK−XCL1 was upregulated in PARK7+NK−C4 (**Figure 3D**), NCR1 was upregulated in UBE2N+NK−C1 (**Figure 3E**), NCR3 was upregulated in PARK7+NK−C4 (**Figure 3E**), TLR9 was upregulated in PARK7+NK−C4 (**Figure 3E**), KIR3DL1 was upregulated in UBE2V1+NK−C2 (**Figure 3E**), LILRB1 was upregulated in UBE2V1+NK−C2 (**Figure 3E**), LILRB2 was upregulated in DYNC1H1+NK−C3 (**Figure 3E**), KLRG1 was upregulated in Non−Aggre−NK−C6 (**Figure 3E**), CEACAM1 was upregulated in PARK7+NK−C4 (**Figure 3E**), CD96 was upregulated in DYNC1H1+NK−C3 (**Figure 3E**), TIGIT was upregulated in PARK7+NK−C4 (**Figure 3E**), LAG3 was upregulated in PARK7+NK−C4 (**Figure 3E**).

### Pseudotime analysis for aggrephagy-mediated myeloid cells

**Figure 4:**
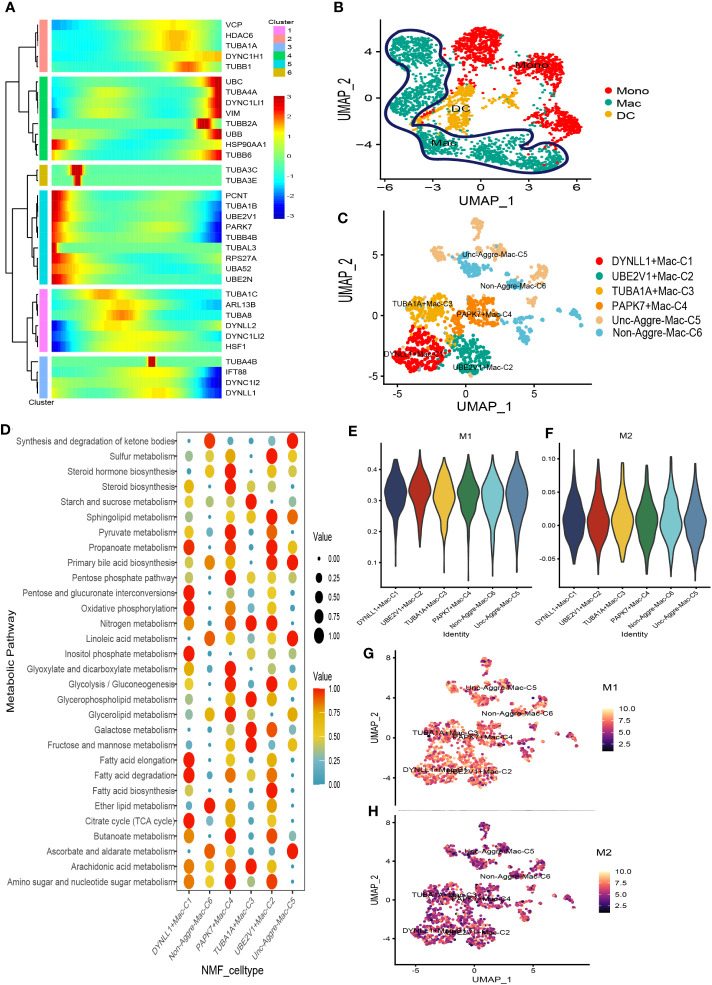
Pseudotime analysis for aggrephagy-mediated Myeloid cells. **(A)** Pseudotime analysis reveals the role of aggrephagy-related genes for Myeloid cells (3167 cells). **(B)** Three subgroups of Myeloid cells were obtained by re-clustering annotation based on UMAP analysis. **(C)** The UMAP view and clustering analysis identifying six aggrephagy-related Mac clusters. **(D)** Bubble map showing significantly different activity of metabolic signaling pathway among the six aggrephagy-related Mac clusters. **(E)** The score of the six aggrephagy-related Mac clusters in M1 type macrophages. **(F)** The score of the six aggrephagy-related Mac clusters in M2 type macrophages. **(G)** UMAP plots of the six aggrephagy-related Mac clusters in M1 type macrophages. **(H)** UMAP plots of the six aggrephagy-related Mac clusters in M2 type macrophages.

There was a total of 3167 cells in the Myeloid cell type. Based on Myeloid cell type, the PCA analysis was carried out on the highly variable genes, the Elbow algorithm to carry out the Standard Deviation based on the highly variable genes (**Supplementary Figure S5**). Using UMAP reduction analysis, the 3167 cells in the Myeloid cell type could be clustered into eleven and twelve cell clusters (**Supplementary Figure S5**). Based on the aggrephagy-related genes, six clusters were identified using Monocle analysis (**Figure 4A**), including Cluster 1 (VCP, HDAC6, TUBA1A, DYNC1H1, and TUBB1), Cluster 2 (UBC, TUBA4A, DYNC1LI1, VIM, TUBB2A, UBB, HSP90AA1, and TUBB6), Cluster 3 (TUBA3C and TUBA3E), Cluster 4 (PCNT, TUBA1B, UBE2V1, PARK7, TUBB4B, TUBAL3, RPS27A, UBA52, and UBE2N), Cluster 5 (TUBA1C, ARL13B, TUBA8, DYNLL2, DYNC1LI2, and HSF1), Cluster 6 (TUBA4B, IFT88, DYNC1I2, and DYNLL1). Three subgroups of Myeloid cells were obtained by re-clustering annotation using UMAP reduction analysis, including Mono (monocytes) cell, Macrophages (MAC) cell, and Dendritic cell (DC) cell (**Figure 4B**). Further, we displayed the global view of the expression pattern for marker genes of Mono (monocytes) cell and Macrophages (MAC) cell in **Supplementary Figure S5**. In addition, the results of UMAP reduction analysis indicated that the NMF cell types were clustered into six aggrephagy-related Mac clusters, including DYNLL1+Mac-C1, UBE2V1+Mac-C2, TUBA1A+Mac-C3, PAPK7+Mac-C4, Unc-Aggre-Mac-C5, and Non-Aggre-Mac-C6 (**Figure 4C**). The number of ligand-receptor links among the aggrephagy-related Mac clusters was computed by Cell-Chat analysis (**Supplementary Figure S5**). Ulteriorly, we used scMetabolism package to assess the correlation between the aggrephagy-related Mac clusters and metabolic pathways, and we could intuitively see the differences in metabolic pathways of each aggrephagy-related Mac cluster from the bubble map (**Figure 4D**). To identify M1/M2 type cells, we scored related genes, suggesting that M1 type macrophages were more active in AML (**Figures 4E–H**).

### Prognostic analysis of the aggrephagy-related cell clusters for acute myeloid leukemia

**Figure 5:**
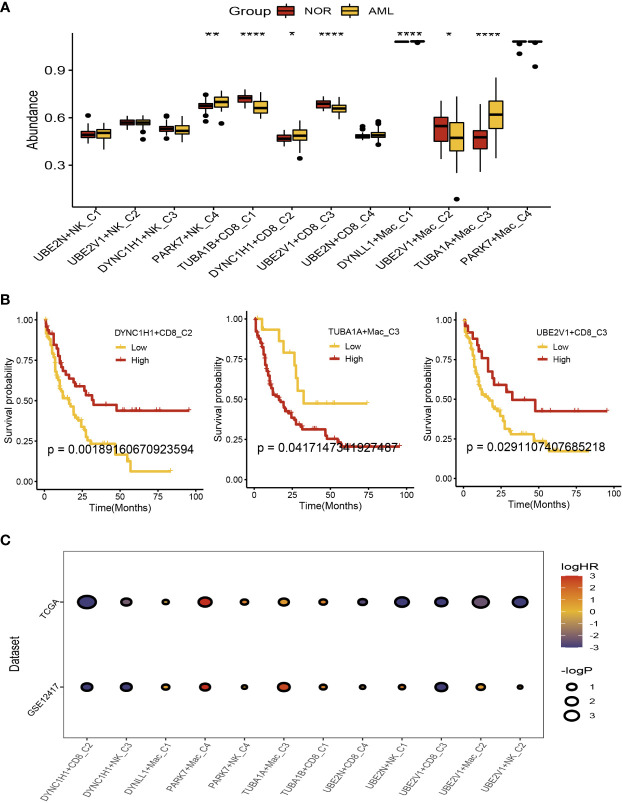
Prognostic analysis of the aggrephagy-related cell clusters for acute myeloid leukemia. **(A)** Comparison for the abundance of the aggrephagy-related cell clusters between normal and AML samples from GSE63270 dataset. **(B)** Kaplan-Meier curves for DYNC1H1+CD8^+^ T−C2, TUBA1A+Mac-C3, and UBE2V1+CD8^+^ T−C3. **(C)** Comparison for the survival rates of AML patients in GSE12417 and TCGA-LAML cohorts among the aggrephagy-related cell clusters. *P < 0.05; **P < 0.01; ****P < 0.0001.

There were 104 normal and acute myeloid leukemia (42 populations and 62 leukemic populations) samples included in GSE63270 dataset, we compared the abundance of UBE2N+NK-C1, UBE2V1+NK-C2, DYNC1H1+NK-C3, PARK7+NK-C4, TUBA1B+CD8^+^ T−C1, DYNC1H1+CD8^+^ T−C2, UBE2V1+CD8^+^ T−C3, UBE2N+CD8^+^ T−C4, DYNLL1+Mac-C1, UBE2V1+Mac-C2, TUBA1A+Mac-C3, and PAPK7+Mac-C4 between normal and AML samples (**Figure 5A**). The results indicated that the higher abundance of PARK7+NK-C4, DYNC1H1+CD8^+^ T−C2, DYNLL1+Mac-C1, and TUBA1A+Mac-C3 were observed in the AML samples, while the higher abundance of TUBA1B+CD8^+^ T−C1, UBE2V1+CD8^+^ T−C3, and UBE2V1+Mac-C2 were observed in the normal samples (**Figure 5A**). Based on the differentially expressed genes (DEGs) generated by the DYNC1H1+CD8+ T−C2, TUBA1A+Mac-C3, and UBE2V1+CD8+ T−C3, the prognostic models were established using TCGA-LAML cohort, the poor prognosis was observed in patients with higher level of DYNC1H1+CD8^+^ T−C2, lower level of TUBA1A+Mac-C3, and higher level of UBE2V1+CD8^+^ T−C3 (**Figure 5B**). GSVA was used for calculating the aggrephagy-related score, the prognosis of the AML patients in the GSE12417 and TCGA-LAML cohorts were further evaluated as displayed in **Figure 5C**. We found that the survival rates of AML patients in GSE12417 and TCGA-LAML cohorts were significantly different among DYNC1H1+CD8^+^ T−C2, DYNC1H1+NK-C3, DYNLL1+Mac-C1, PAPK7+Mac-C4, PARK7+NK-C4, TUBA1A+Mac-C3, TUBA1B+CD8^+^ T−C1, UBE2N+CD8^+^ T−C4, UBE2N+NK-C1, UBE2V1+CD8^+^ T−C3, UBE2V1+Mac-C2, and UBE2V1+NK-C2 (**Figure 5C**).

### Immunotherapy analysis of the aggrephagy-related cell clusters for acute myeloid leukemia

**Figure 6:**
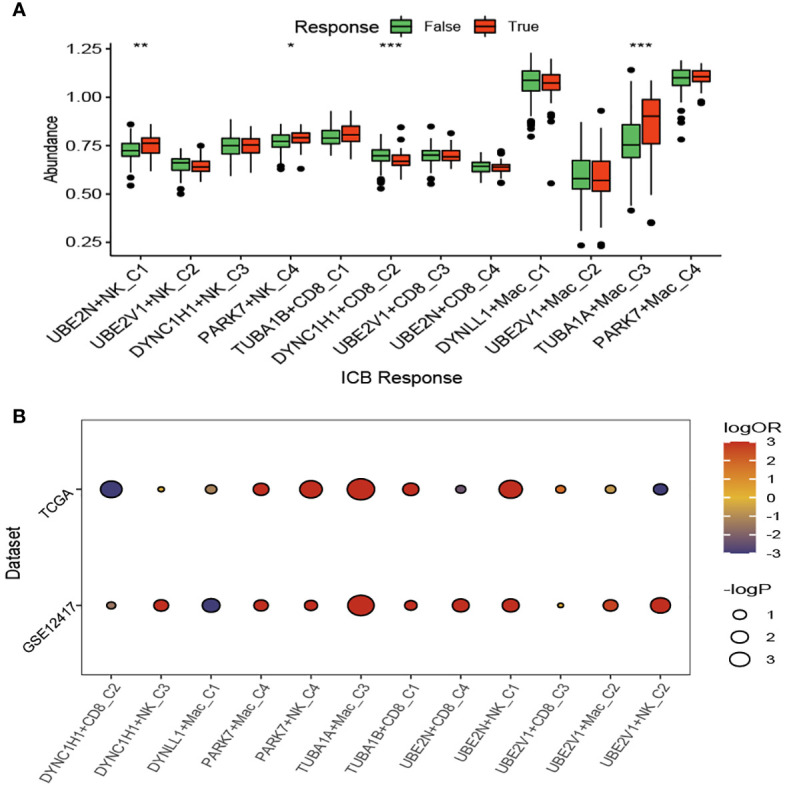
Immunotherapy analysis of the aggrephagy-related cell clusters for acute myeloid leukemia based on TIDE algorithm. **(A)** Comparison for the response status of immune checkpoint blockade therapy for patients with AML among the aggrephagy-related cell clusters. **(B)** Comparison for the OR rates of AML patients in GSE12417 and TCGA-LAML cohorts among the aggrephagy-related cell clusters. *P < 0.05; **P < 0.01; ****P < 0.0001.

We compared the response status (False or True) of immune checkpoint blockade therapy for patients with AML among the aggrephagy-related cell clusters (UBE2N+NK-C1, UBE2V1+NK-C2, DYNC1H1+NK-C3, PARK7+NK-C4, TUBA1B+CD8^+^ T−C1, DYNC1H1+CD8^+^ T−C2, UBE2V1+CD8^+^ T−C3, UBE2N+CD8^+^ T−C4, DYNLL1+Mac-C1, UBE2V1+Mac-C2, TUBA1A+Mac-C3, and PAPK7+Mac-C4) using TIDE algorithm (**Figure 6A** and **Supplementary Figure S6**). For the AML patients with True response status, the abundance of UBE2N+NK-C1, PARK7+NK-C4, and TUBA1A+Mac-C3 was higher, while the abundance of DYNC1H1+CD8^+^ T−C2 was lower (**Figure 6A**). We found that the OR rates of AML patients in GSE12417 and TCGA-LAML cohorts were significantly different among DYNC1H1+CD8^+^ T−C2, DYNC1H1+NK-C3, DYNLL1+Mac-C1, PAPK7+Mac-C4, PARK7+NK-C4, TUBA1A+Mac-C3, TUBA1B+CD8^+^ T−C1, UBE2N+CD8^+^ T−C4, UBE2N+NK-C1, UBE2V1+CD8^+^ T−C3, UBE2V1+Mac-C2, and UBE2V1+NK-C2 (**Figure 6B**).

**Figure 7:**
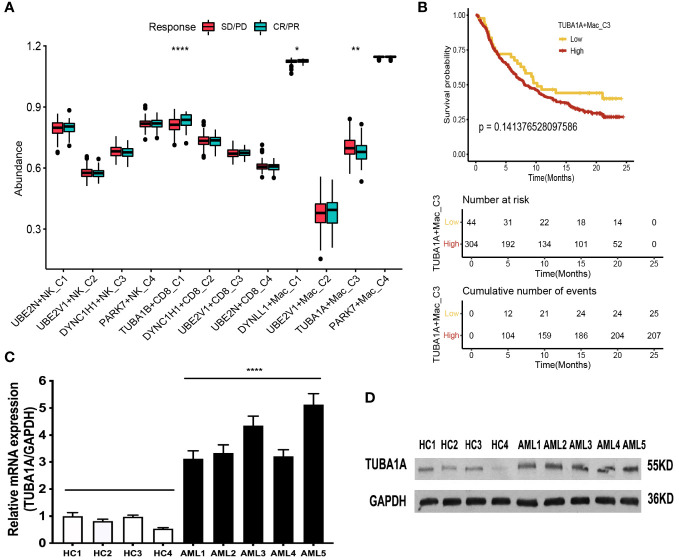
Immunotherapy analysis of the aggrephagy-related cell clusters for AML based on public dataset. **(A)** Comparison for the response status of immune checkpoint blockade therapy for patients with AML among the aggrephagy-related cell clusters. **(B)** Kaplan-Meier curve for TUBA1A+Mac-C3. **(C)** The TUBA1A mRNA expression is upregulated in bone marrow cells of AML patient. The levels of TUBA1A mRNA and GAPDH mRNA as control in bone marrow cells of five AML patients and four health people by real-time PCR. Data are expressed as mean ± SD. (**** P <0.0001). **(D)** The TUBA1A protein expression is upregulated in bone marrow cells of AML patient. The levels of TUBA1A protein in bone marrow cells of five AML patients and four health people as control by Western-blot. *P < 0.05; **P < 0.01; ****P < 0.0001.

In addition, we compared the response status (SD/PD or CR/PR) of immunotherapy for patients with AML among the aggrephagy-related cell clusters (UBE2N+NK-C1, UBE2V1+NK-C2, DYNC1H1+NK-C3, PARK7+NK-C4, TUBA1B+CD8^+^ T−C1, DYNC1H1+CD8^+^ T−C2, UBE2V1+CD8^+^ T−C3, UBE2N+CD8^+^ T−C4, DYNLL1+Mac-C1, UBE2V1+Mac-C2, TUBA1A+Mac-C3, and PAPK7+Mac-C4) based on public dataset (**Figure 7A**). For the AML patients with CR/PR response status, the abundance of TUBA1B+CD8^+^ T−C1 and DYNLL1+Mac-C1 was higher, while the abundance of TUBA1A+Mac-C3 was lower (**Figure 7A**). We also found that AML patients with low abundance of TUBA1A+Mac-C3 may have the better prognosis (**Figure 7B**). In addition, we observed that the expression of TUBA1A was upregulated in bone marrow cells of AML patient both in mRNA (**Figure 7C**) and protein (**Figure 7D**) levels.

## Discussion

Leukemia is a kind of hematologic malignant disease with hematopoietic stem cell clonal proliferation. Clonal leukemia cells proliferate and accumulate in bone marrow and other normal hematopoietic tissues, inhibit hematopoietic function, and penetrate into other non-hematopoietic tissues and organs through blood circulation, resulting in organ failure and poor prognosis. The clinical manifestations of AML include anemia, bleeding, infection fever and other symptoms. AML is a common type of leukemia, accounting for 80% of acute leukemia, with a high incidence in children (61). Patients with AML tend to die within one year of diagnosis, with a high mortality rate (62). The pathogenesis of AML is complex and diverse, including chemical substances, radioactive substances, genetic factors, gene mutations, abnormal signaling pathways, epigenetic regulation, leukemia microenvironment or immune imbalance. Autophagy is a catabolic process of intracellular substances mediated by lysosome, which has a bidirectional effect in AML. Autophagy can remove abnormal organelles, reduce the accumulation of harmful substances, and effectively prevent cell cancer. However, autophagy can also enable AML cells to obtain various substances and energy, which can help malignant cells to fight against the lack of nutrition and energy caused by their own high metabolism, and promote the growth and proliferation of AML cells. The autophagy levels in different stages of AML were different. How to regulate the progression of AML, remove AML cells and improve the therapeutic effect by regulating autophagy level is the focus of AML prevention and treatment.

The survival and apoptosis of immune cells, the expression of immunomodulators and the change of tumor microenvironment (TME) all affect the occurrence and development of AML (25). Immune cells monitor abnormal cells in the body and play an immune effect to eliminate them (63). For example, nature killer (NK) cells recognize and kill tumor cells by mediating cytotoxic effects (64). Tumor cells can evade immune recognition and attack by modifying their own surface antigens and changing the microenvironment around tumor tissue, that is, immune escape of tumor. The occurrence of AML is also closely related to immune escape. By changing the activity of immune cells or regulating the expression of immune molecules, the function of immune cells is affected, thus achieving immune escape of AML cells (65). It can effectively treat AML by inhibiting the cell immune microenvironment and enhancing the immune response (66). To elucidate the relationship between the occurrence of AML and the immune response is of great significance for the development of immunotherapy in patients with AML. In this study, we identified 11 specific cell types, including T cell, Natural Killer (NK) cell, Common Myeloid Progenitor (CMP) cell, Myeloid cell, Granulocyte Monocyte Progenitor (GMP) cell, Megakaryocyte Erythroid Progenitor (MEP) MEP, Promonocyte (Promono) cell, Plasma cell, Hematopoietic Stem Cell (HSC) cell, B cell, and Erythroid cell for AML. Four subgroups of T cells were obtained by re-clustering annotation using t-SNE dimension reduction analysis, including CD8+ T cell, CD4+ T cell, natural killer (NK) cells, and Regulatory T (Treg) cells. NK cell type could be clustered into eleven cell clusters. As for Myeloid cells, three subgroups of Myeloid cells were obtained by re-clustering annotation using UMAP reduction analysis, including Mono (monocytes) cell, Macrophages (MAC) cell, and Dendritic cell (DC) cell. Our study identified some specific cell subtypes of AML, which will provide some reference value for exploring the TME of AML.

Transcription factors (TFs) are involved in the formation of transcription initiation complexes that affect transcription processes and thus downstream gene expression (67). AML contains many abnormal genes, some of which directly affect the expression of TFs, and some indirectly affect the combination of transcription factors and regulatory regions to play a role (68). In addition, some TFs play a role in stem cell maintenance, differentiation and maturation of hematopoietic stem progenitor cells, and abnormal expression of these TFs can lead to hematopoietic malignant transformation. Herein, we found that TFs of LGALS9, TIGIT, BTLA, and CTLA4 were upregulated in the TUBA1B+CD8^+^ T−C1, TFs of IL10 and CD160 were upregulated in the DYNC1H1+CD8^+^ T−C2, ADORA2A was upregulated in the UBE2V1+CD8^+^ T−C3, BTN3A1, BTN3A2, CD274, CD247, SLAMF7, LAG3, and PDCD1 were upregulated in the UBE2N+CD8^+^ T−C4, BTN2A2 and LAIR1 were upregulated in the Non−Aggre−CD8^+^ T−C6. AML is a highly heterogeneous and aggressive hematological malignancy resulting from clonal expansion of malignant hematopoietic progenitor cells in the bone marrow. Its incidence increases with age and its prognosis is poor. A variety of cytogenetic and molecular genetic abnormalities affect signaling pathways, transcription, and epigenetic regulators that induce AML. Studies have shown that various recurrent gene mutations can directly affect the expression of TFs or indirectly change the binding of TFs to regulatory regions, resulting in abnormalities of transcriptional regulatory networks (TRNs), leading to a large number of cloning and proliferation of myeloid precursor cells and stagnating in different stages of hematopoietic differentiation. The fine regulation of TFs such as TIGIT (69), BTLA (70), CTLA4 (71), IL10 (72), CD274 (73) is crucial in hematopoietic regulation and cell fate determination. Abnormal expression of these TFs can interfere with normal hematopoietic differentiation and cause the occurrence of AML. Our study provided new insights into the regulatory mechanisms of related TFs in cell subtypes of AML.

## Data availability statement

The original contributions presented in the study are included in the article/**Supplementary Material**. Further inquiries can be directed to the corresponding authors.

## Author contributions

Our study revealed the significance of aggrephagy-related patterns in tumor microenvironment, prognosis, and immunotherapy for AML. All authors contributed to the article and approved the submitted version.

## Supplementary material

Supplementary Figure 1 Dimensionality reduction of single cell for acute myeloid leukemia. **(A, B)** The sequencing depth and the number of genes for single cell from three normal samples and ten patients with acute myeloid leukemia. **(C)** Detection of the highly variable genes across the cells in volcano plot, the top 10 genes were marked out. **(D)** PCA plot of scRNA-seq samples from 13 samples and the Standard Deviation of 1-20 PCs using ElbowPlot algorithm. **(E)** t-SNE and UMAP dimension reduction analysis identifying a total of 18 cell subsets.

Supplementary Figure 2 Dot plot showing the average and percentage expression of well-defined marker genes in different cell subsets. The color represented the average expression level of the marker genes. The diameter of the dots denoted the fractional expression.

Supplementary Figure 3 UMAP reduction analysis and cell–cell communication analysis for aggrephagy-mediated T cells.

Supplementary Figure 4 UMAP reduction analysis and cell–cell communication analysis for aggrephagy-mediated NK cells.

Supplementary Figure 5 UMAP reduction analysis and cell–cell communication analysis for aggrephagy-mediated Myeloid cells.

Supplementary Figure 6 Immunotherapy analysis of the aggrephagy-related cell clusters for acute myeloid leukemia using TIDE algorithm.
