# Supplementary material for: Aggrephagy-related patterns in tumor microenvironment, prognosis, and immunotherapy for acute myeloid leukemia: a comprehensive single-cell RNA sequencing analysis
Source: Front Oncol. 2023 Jul 17;13:1195392. doi: 10.3389/fonc.2023.1195392 (PMC10393257; doi:10.3389/fonc.2023.1195392)

Supplemental Figure S1


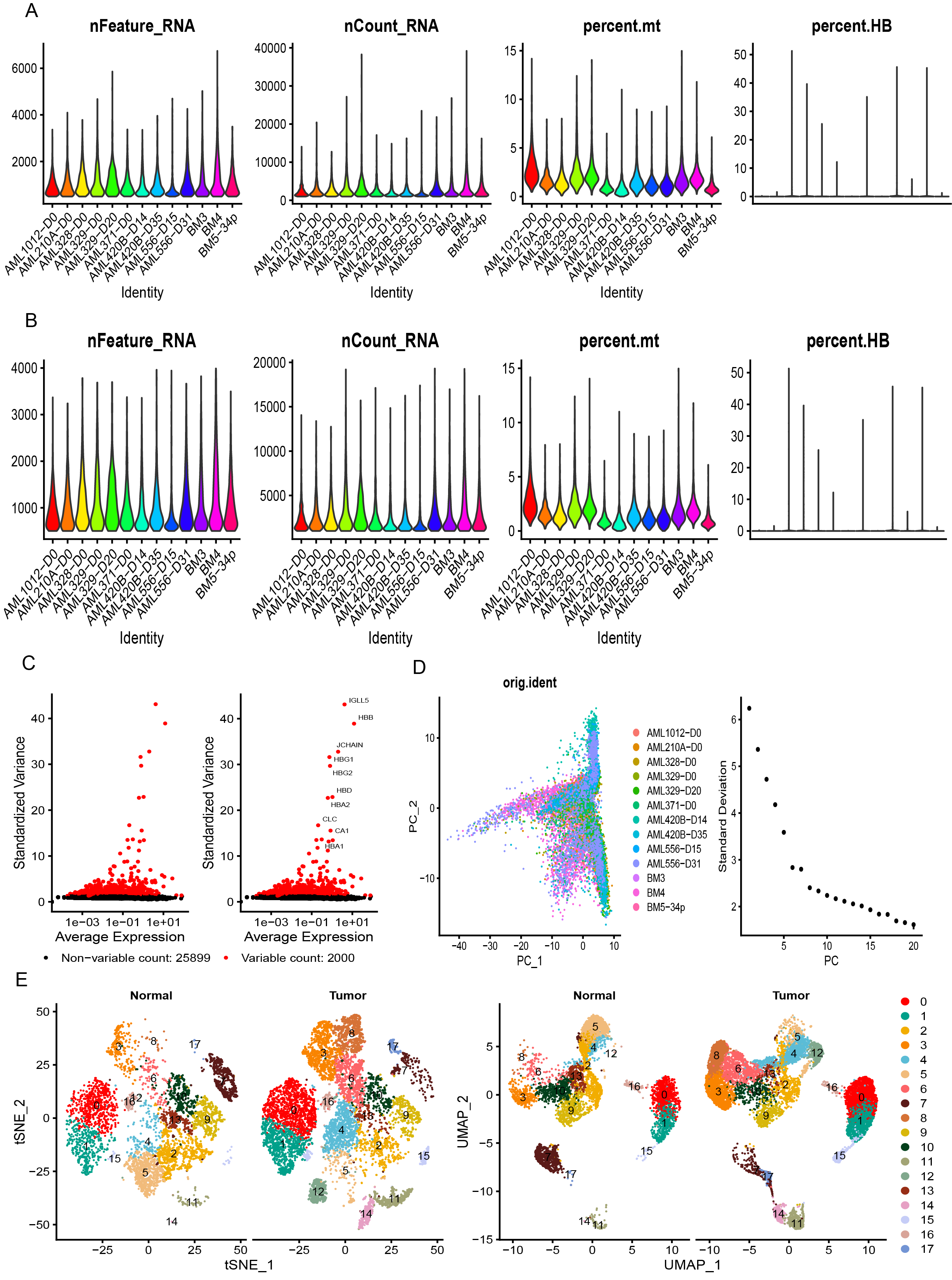


Supplemental Figure S2


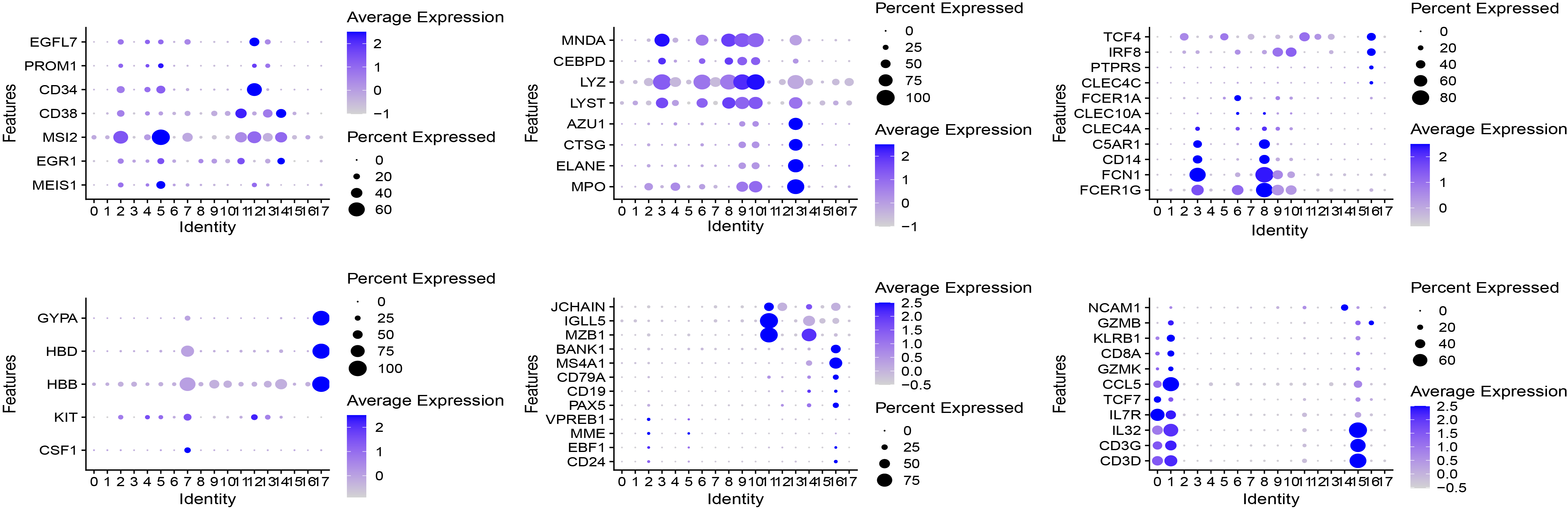


Supplemental Figure S3


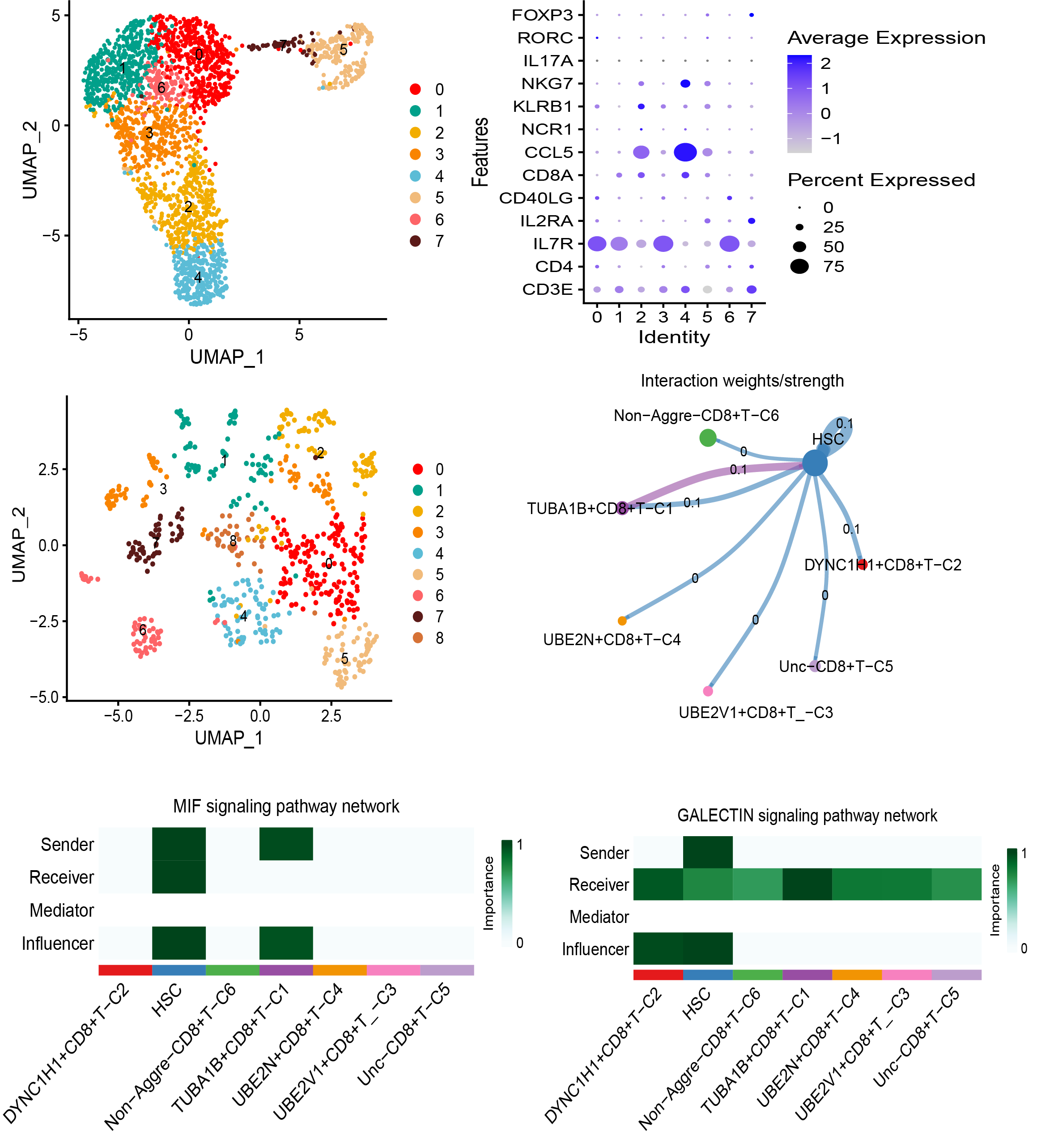


Supplemental Figure S4


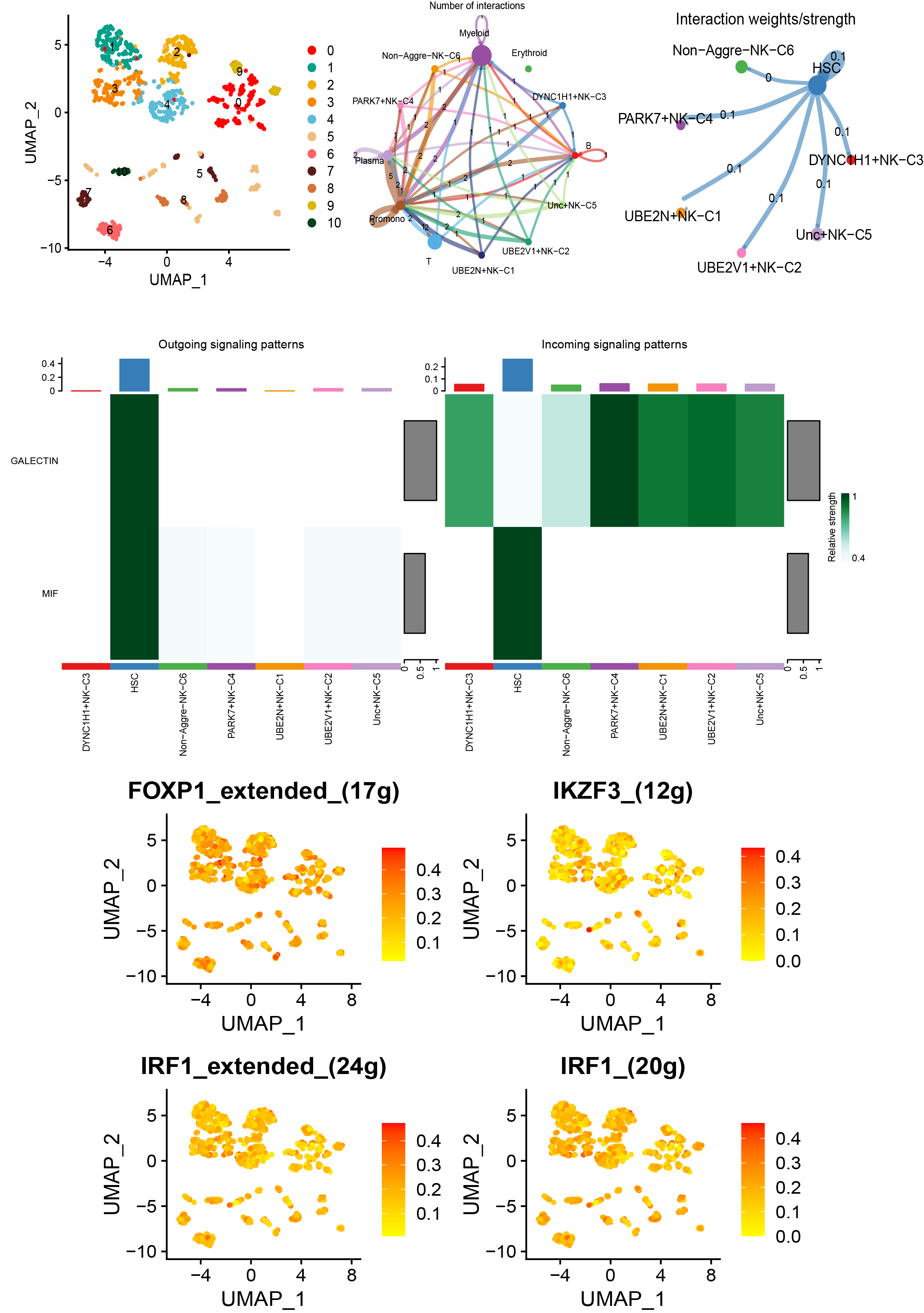


Supplemental Figure S5


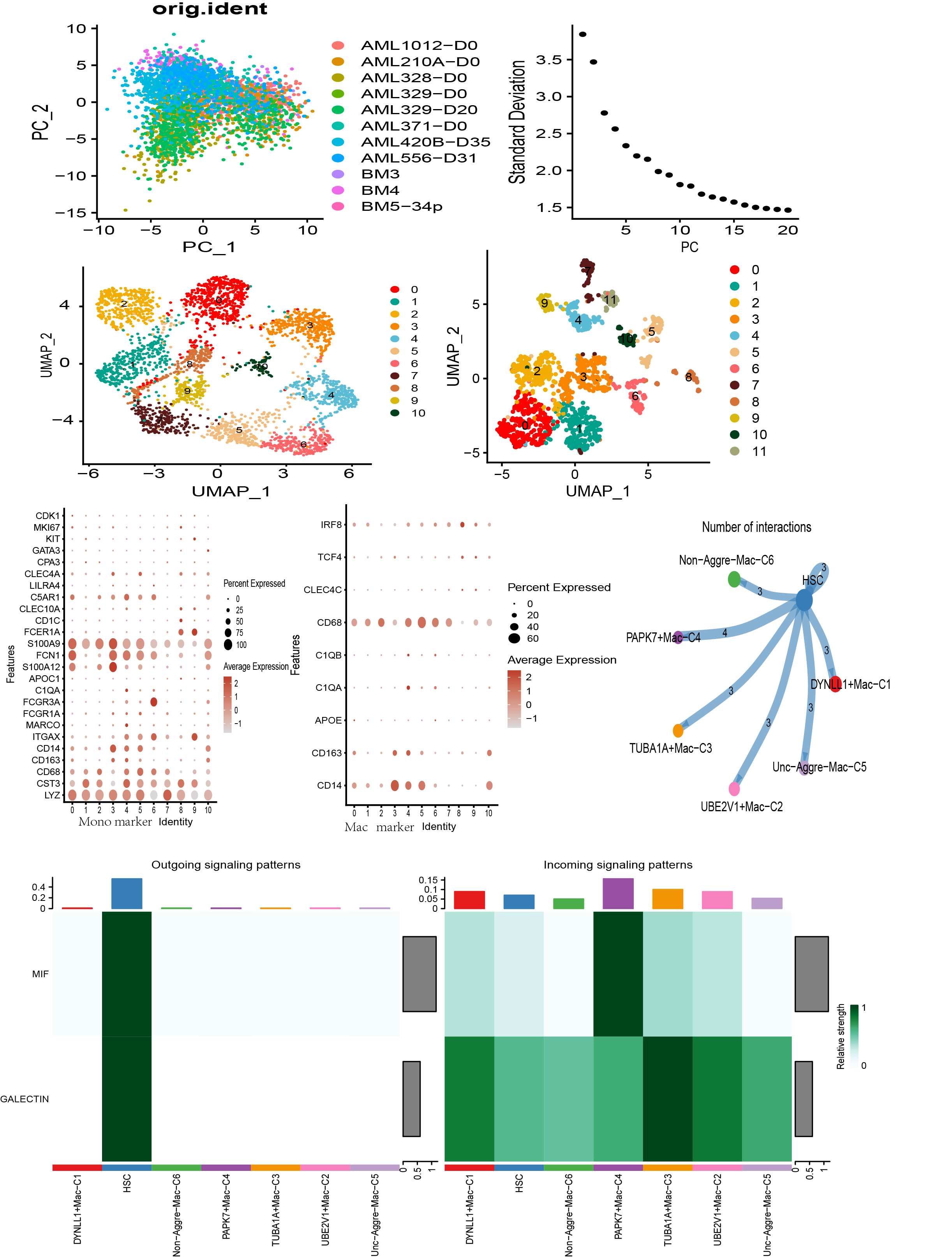


Supplemental Figure S6


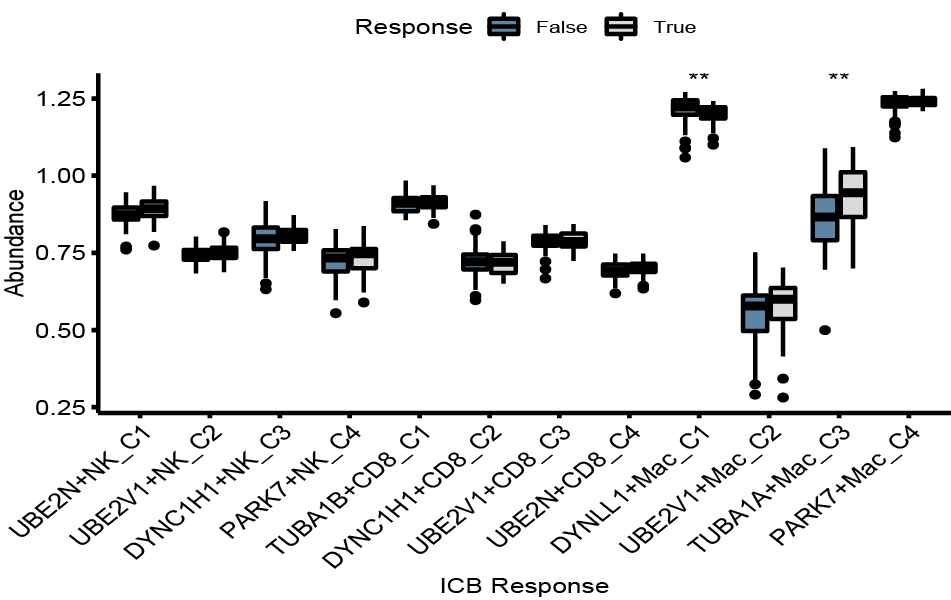

Supplement: Supplementary Figure 1 — Dimensionality reduction of single cell for acute myeloid leukemia. (A, B) The sequencing depth and the number of genes for single cell from three normal samples and ten patients with acute myeloid leukemia. (C) Detection of the highly variable genes across the cells in volcano plot, the top 10 genes were marked out. (D) PCA plot of scRNA-seq samples from 13 samples and the Standard Deviation of 1-20 PCs using ElbowPlot algorithm. (E) t-SNE and UMAP dimension reduction analysis identifying a total of 18 cell subsets. [file Image_1.docx]
